# Downward social comparison positively promotes altruism: the multi-mediating roles of belief in a just world and general life satisfaction

**DOI:** 10.3389/fpsyg.2024.1386860

**Published:** 2024-06-20

**Authors:** Yunjun Hu, Guanyu Cui, Linxi Jiang, Xiaoyu Lan

**Affiliations:** ^1^Department of Students' Affairs, Wenzhou University of Technology, Wenzhou, China; ^2^Research Center for Psychology and Behavior, Wenzhou University, Wenzhou, China; ^3^Department of Psychology, School of Education, Wenzhou University, Wenzhou, China; ^4^PROMENTA Research Center, Department of Psychology, University of Oslo, Oslo, Norway

**Keywords:** downward social comparison, altruism, general belief in a just world, personal belief in a just world, general life satisfaction

## Abstract

**Introduction:**

This study examined the underlying mechanism of the relationship between downward social comparison (DSC) and altruism through two conceptually important but rarely studied factors: belief in a just world (BJW) and general life satisfaction (GLS).

**Methods:**

The study utilized a two-time-point design, spaced 2 months apart, with a sample of 1,764 college students from China. The study measured DSC, altruism, BJW, and GLS.

**Results:**

The findings revealed a significant positive predictive effect of DSC on college students’ altruism and belief in a just world (BJW). Notably, GLS emerged as a multi-mediating factor in this relationship. The study also revealed that both subscales of BJW, namely personal belief in a just world (PBJW) and general belief in a just world (GBJW), played distinct roles in the mediation model. PBJW exhibited a stronger mediating effect, suggesting that DSC can foster individual altruism and BJW. Particularly, BJW was identified as enhancing GLS, subsequently promoting altruistic behavior.

**Conclusion:**

This study contributes to the existing literature on social comparison by shedding light on the relationship between DSC, altruism, and the mediating role of belief in a just world and GLS. The findings underscore the potential for promoting altruistic behavior among college students through interventions targeting beliefs in a just world, especially at the personal level, and enhancing GLS.

## 1 Introduction

Altruism is an important sociological topic that reflects a range of behaviors that benefit others at a personal cost (Giancola et al., 2022). As a higher-level prosocial behavior, altruism is significant for both individuals and society. For individuals, altruism is not only conducive to happiness, health, and longevity (Post, 2005), but also brings more interpersonal benefits, such as making individuals look more attractive and having more advantages in partner selection (Farrelly, 2013). In terms of society, Batson (1991) defined altruism as “a motivated state with the ultimate goal of increasing another’s welfare,” and altruism has a positive impact on individuals, such as obtaining social rewards (Batson et al., 1988), promoting group trust, and establishing good cooperative relationships (Barclay, 2004).

On one hand, some studies believe that the desire to help others selflessly is also the true motive for human behavior (Sznycer et al., 2019) and altruism has been confirmed to be the basis of neurophysiological mechanisms (Morishima et al., 2012; Weng et al., 2013), altruism does not always occur. On the other hand, more researchers focused on psychological altruism (Batson, 2010). Batson (1991) deemed that “empathic concern produces altruistic motivation, not egoistic alternatives,” raised the empathy-altruism hypothesis; later, Batson (2010) verified the hypothesis through more than 30 experiments. The evolutionary model of altruism believes that the influence of an “interactive environment” is the most basic factor in understanding the evolution of altruism (Fletcher and Doebeli, 2009), and altruism can be subjectively selected under the influence of an interpersonal environment. For example, Barclay and Willer (2007) found that when people benefit from the choice of cooperative partnerships, they tend to actively compete to be more generous than others. Correspondingly, the most generous people are chosen more often as partners. As a result, people sometimes pretend to be generous when conditions permit generosity.

Socialization and feedback from social interactions with others are factors that influence altruism (Warneken and Tomasello, 2009). Thus, we considered the correlation between social comparison and altruism. Social comparisons include both upward and downward comparisons. Previous studies have suggested that downward social comparison (DSC) has more positive effects on prosocial behavior than upward comparison (Ye et al., 2021). Therefore, this study examines the effect of DSC on altruism. Some studies have found that social comparison affects individuals’ beliefs in a just world (BJW) (Pan and Zhao, 2023) and general life satisfaction (GLS) (Olivos et al., 2021), whereas others have separately determined the significant direct or indirect effects of individuals’ BJW and GLS on altruism (Bierhoff et al., 1991; Ummet et al., 2015; Quan et al., 2022). However, few studies have investigated the multi-mediating role of the two variables, BJW and GLS, between DSC and altruism. This study intends to incorporate them into the structural equation model and verify them. Related studies on BJW have found that personal belief in a just world (PBJW) and general belief in a just world (GBJW) often play different roles independently (Dalbert, 1999), Therefore, their effects in the model will be examined separately in this study.

### 1.1 DSC and altruism

Festinger (1954) proposed that human social behavior stems from the drive for self-evaluation and the need for such evaluation with others, which is innate and varies in the absence of a clear ranking of relevant behavioral standards. When individuals are in a low-certainty environment, they gain a sense of control by comparing themselves with others in the absence of objective criteria or perceived irrelevance. Social comparison involves a series of social and cognitive activities. People evaluate, improve, or enhance themselves by comparing their thoughts, feelings, and behaviors with those of others (Stapel and Koomen, 2001) and influence individuals’ social behaviors or emotions. The results of such influence are related to the direction of social comparison. In a related study on classroom behavior, researchers found that students prefer to compare their performance upwards, especially those who perform better than themselves but are like themselves in terms of related or unrelated attributes. This upward comparison affects students’ academic performance and self-concept (Dijkstra et al., 2008).

A related study on cancer patients showed that cancer patients with low self-esteem and a low ability to control their symptoms and disease may have a negative reaction to both downward and upward social comparisons (Buunk et al., 2007). Studies suggest that social comparison has a significant effect on altruism, which is related to the direction of comparison. Compared to upward social comparison, DSC has more positive effects on prosocial behavior (Ye et al., 2021). Through experiments, Klein (2003) found that feedback, especially comparative feedback, affects the degree to which recipients are willing to help others. Participants who received positive feedback on the task were more likely to provide useful hints to another participant on a follow-up task than those who received negative feedback, particularly in situations in which feedback was provided to other participants or the average person. Schlosser and Levy (2016) also found that people who make downward comparisons are more likely to view donations as a way to express altruistic values and are more willing to give donations than those who make upward comparisons. Based on this discussion, we propose the following hypothesis.

*Hypothesis 1 (H1)*: DSC has a positive and significant effect on altruism.

### 1.2 Mediating role of BJW

The concept of a just world was first proposed by Lerner (1965); individuals believe that the world in which they live is stable, orderly, and just, in which everyone will receive fair treatment, enjoy their employment rights, and reap what they have sown. BJW is a basic need for individuals, helping them rebuild their sense of fairness, enhancing their sense of control, reducing anxiety, and positively impacting their adaptive function (Lerner and Miller, 1978). BJW is generally divided into two fields: PBJW and GBJW. PBJW refers to the belief that individuals deem that they are treated as fairly as they deserve, while GBJW refers to the belief that individuals deem the world fair and that everyone is treated as they deserve (Dalbert, 1999).

According to attribution theory, socioeconomic status is closely related to people’s BJW, and those with higher socioeconomic status are more likely to support BJW; they must view the world fairly in order to believe that they have achieved success on their own. On the contrary, people with lower socio-economic status are less likely to support BJW, maintaining their self-esteem by blaming society rather than their own reasons. In this process, the essence of how wealthy people view the world is a downward comparison regarding wealth, which also leads to and enhances their BJW (Kehl, 2010). Previous studies have further proven this theory (Smith and Green, 1984; Smith, 1985).

The BJW has a positive social significance; the more individuals believe that the world is just, the more willing they are to engage in prosocial behaviors (Guo et al., 2022). BJW directly promotes altruism by increasing willingness to help others (Bierhoff et al., 1991). It promotes altruism through indirect variables. Individuals are happy to engage in acts of charitable giving in the community only if they are confident that their cooperative and caring attitude of kindness will not be taken advantage of Guo et al. (2022).

The GBJW and PBJW often function independently. Research shows that PBJW can be reliably distinguished from GBJW as it can independently and positively predict subjective well-being and self-esteem, and this positive effect is not affected by GBJW or good self-perception (Dalbert, 1999). Moreover, PBJW is positively correlated with job and psychological satisfaction (Otto et al., 2009). Compared with PBJW, the expression of GBJW is more sensitive to contextual changes (Alves et al., 2018). For example, in the context of collectivist cultures, GBJW is more recognized and independently predicts the mental resilience of the three samples (Wu et al., 2011). GBJW has also been found to protect the mental health of individuals facing major social disasters. High levels of GBJW can help individuals reduce negative emotions and increase positive emotions (Wang J. et al., 2021).

BJW involves the perception and understanding of the social environment. Different social comparison directions influence the effects of BJW. Bégue (2005) measured the self-esteem of participants in upward or downward comparison social situations and found that when faced with DSC, a high PBJW can help individuals maintain self-esteem under high self-efficacy. In a study related to relative deprivation, Pan and Zhao (2023) demonstrated that upward social comparison weakens PBJW, resulting in them not believing in future promises and adopting more short-sighted immediate reward strategies. In conclusion, DSC may have a more positive effect on BJW. Therefore, we propose the following hypotheses:

*Hypothesis 2 (H2)*: GBJW partially mediates the association between DSC and altruism.

*Hypothesis 3 (H3)*: PBJW partially mediates the association between DSC and altruism.

*Hypothesis 4 (H4)*: GBJW plays a stronger mediating role than the PBJW in this study.

### 1.3 Mediating role of GLS

The GLS is an important component of subjective well-being and refers to a person’s overall assessment of his or her quality of life according to selected criteria (Shin and Johnson, 1978). GLS is a key factor that affects an individual’s mental health and social relationships. Fuller-Iglesias (2015) found that the higher the satisfaction with social support, the lower the pressure and fewer the depressive symptoms. Longstreet and Brooks’s (2017) study shows that GLS has a significant impact on generalized network addiction and social media addiction, when GLS, Internet addiction, and specific social media addiction are reduced. Bozoglan et al. (2013) found that GLS was significantly associated with self-esteem, loneliness, and Internet addiction. Moreover, it indirectly affects internet addiction through self-esteem and loneliness. Similar findings have been made in studies related to altruism: Ummet et al. (2015) found that the GLS level of college students can predict altruistic behavior, and with the improvement of GLS, their helping behavior toward others will also increase. Valentine et al. (2011) found that when employees have a high degree of career satisfaction, a company’s commitment to positive corporate ethical values significantly improves the degree to which employees help their colleagues.

Generally, life satisfaction involves cognitive judgment and life evaluation. Individuals’ GLS is affected only by what they see in others if they have a strong tendency to compare themselves with others (Buunk et al., 2007). Social comparisons, especially downward comparisons, are better predictors of GLS than factors such as expectations or comparisons with previous situations. In relevant studies on the elderly population, social comparison is a positive adaptive strategy used by the elderly to maintain their GLS (Frieswijk et al., 2004a). Intragroup social comparison has also been found to be a determinant of GLS across racial and ethnic groups (Davis and Wu, 2014). Studies of college students have also shown that social comparison affects students’ evaluations of their friendships, the quality of their social relationships, and their social lives (Buunk et al., 2007). Upward comparisons may reduce GLS (Antinyan, 2016), whereas downward comparisons may enhance it (Frieswijk et al., 2004b). Based on this, this study proposes the following hypothesis:

*Hypothesis 5 (H5)*: GLS partially mediates the association between DSC and altruism.

### 1.4 The multi-mediating role of BJW and GLS

**Figure 1:**
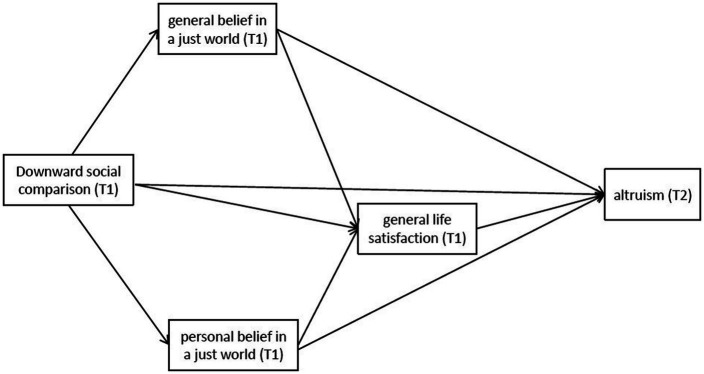
The proposed multi-mediating model.

Regarding the relationship between BJW and GLS, research has shown that BJW is based on people’s belief that the world is just and that they can obtain what they deserve, which is a crucial factor affecting GLS. When BJW increases, GLS tends to increase (Tian, 2019), whereas a decrease in BJW decreases GLS (Chen et al., 2022). Hirshleifer (1987) believed that empathy was the driving factor in maintaining social fairness and justice. Individuals evaluate situations and clarify empathy through social comparisons with others (Buunk et al., 1990). The empathy generated during the comparison process also influence the subsequent behavior of an individual (Rotteveel and Phaf, 2007), which is supported by empirical research. Yu et al.’s (2018) study of 372 Chinese college students showed that BJW could significantly predict subjective well-being. Oishi et al. (2011) proposed that perceived inequity mediates the relationship between income inequality and GLS. Income inequality leads to high social comparison, and high social comparison levels lead to perceived inequity and a lack of trust, resulting in low GLS (Figure 1). Accordingly, we propose the following hypothesis:

*Hypothesis 6 (H6)*: GBJW/PBJW, together with GLS, play a multi-mediating role in the influence of DSC on altruism.

## 2 Materials and methods

### 2.1 Participants and procedure

Convenience samples of 1,764 university students were chosen in this study, including 739 males and 1,025 females from one college in Henan province, China. We distributed 1,900 Sojump online questionnaires and 1,850 responses, including data from two time points, were collected, resulting in an impressive 97.4% response rate. After carefully examining the survey data, 86 responses were excluded due to patent responses or excessive missing values. Consequently, the final analysis included a total of 1,764 valid responses. The inclusion criteria for our sample were students enrolled in this college, while the exclusion criteria included incomplete questionnaire responses or evident response patterns indicating non-serious participation. Participants were selected based on their willingness and ability to provide consistent responses over time. The average age (M_age) was 19.21 years, accompanied by a standard deviation (SD_age) of 1.00 years with the majority falling into the age groups of 18, 19, or 20 years, comprising 22.0, 40.0, and 25.7% of the sample, respectively. Notably, the highest proportion of participants, 40.0%, fell into the 19-year-old group, highlighting the significance of this age cohort in the study. We collected the data from two time points with an interval of 2 months. The study was conducted in 2022, collaborating with local education bureaus and ensuring voluntary participation.

### 2.2 Measures

#### 2.2.1 Altruism

Altruism was measured with the subscale of altruism tendency in Prosocial Tendencies Measure–Revised (PTM-R) (Carlo et al., 2003), with a total of six items, like “I often help even if I do not think I will get anything out of helping,” rated on a 5-point Likert response (1 = “not true at all” to 5 = “true nearly all the time”). Part of the question was scored in reverse before the total score was calculated. PTM-R is a self-report scale that is easily applied and widely used (Xiao et al., 2019; Caetano et al., 2023). Previous researchers have utilized the altruism subscale to determine altruism at the trait level and have confirmed that it has good reliability and validity (Rodrigues et al., 2018; Song et al., 2023). Higher summed scores indicate higher levels of altruism, and the reliability coefficients in both two measures are 0.90.

#### 2.2.2 DSC

We used the scale developed by Van der Zee et al. (2000) to measure DSC. For example, DSC was measured by asking participants “When I see others who are doing worse, I experience fear that my health status will decline.” Participants answered six items on a 5-point Likert scale (1 = “strongly disagree,” 5 = “strongly agree”), and higher summed scores indicated a higher level of DSC. Previous studies have demonstrated that this scale has good reliability and validity (Li et al., 2021). The reliability coefficients of the two measurements in this study are 0.96 and 0.95, respectively.

#### 2.2.3 PBJW/GBJW

The BJW was measured by the Justice Centrality Scale (Dalbert, 1999), with 13 items gauging distress about injustice and satisfaction with one’s fairness (e.g., “Injustice that I caused torment me for a long time,” “There are few things that make me as happy as justice”). A 6-point Likert response scale (1 = “strongly disagree,” 6 = “strongly agree”) was used. The BJW contains two subscales: the PBJW contains seven items to investigate beliefs about a just world in relation to one’s life, and the GBJW contains six items to investigate beliefs about a just world in relation to a wide range of life events. All items are summed to obtain a total score. Higher scores indicate higher levels of BJW. Previous studies have indicated that this scale has good reliability and validity (Ma et al., 2023). For the present study, the reliability coefficients of the two measurements of GBJW are 0.95 and 0.93 respectively, while that of PBJW are 0.97 and 0.96.

#### 2.2.4 GLS

The GLS was measured through a questionnaire consisting of six items (e.g., “I am satisfied with my life,” “The conditions of my life are excellent”), including the 5-item GLS Scale developed by Diener et al. (1985) and a single item for measuring the overall GLS of participants (Andrews and Withey, 1976). Previous studies have confirmed that this scale has good reliability and validity (Leung and Leung, 1992). Considering the overall and unified nature of the research measurement, a 6-point scoring method was used for the questionnaire. Higher scores indicate higher levels of GLS. The reliability coefficients of the two measurements in this study are 0.89 and 0.88, respectively.

### 2.3 Data analysis

Descriptive statistics and correlation analyses were performed using SPSS26.0, while Mplus 8.3 was used to analyze the multi-mediating effects.

### 2.4 Results

#### 2.4.1 Common method bias test

Common method bias refers to the influence bias caused by common method variation, which occurs when using the same scales, test environment and instructions, etc. To control common method deviation in this study, we reversed the expression and coding of some questions and standardized the guidelines. Harman’s single-factor test was used for testing common method bias prior to data analysis. Results showed that the first common factor explained 31.61% of the total variance (i.e., less than the standard of 40%). Therefore, there were no significant common methodological biases in this study.

#### 2.4.2 Descriptive statistics and related analysis

A correlation analysis was conducted to explore the relationship between DSC, GBJW/PBJW, GLS, and altruism measured at two time points (T1 and T2). Table 1 presents the results of the analysis. All variables were significantly correlated with each other. T1 DSC showed a significant positive correlation with T1 GBJW (*r* = 0.05, *p* < 0.05), T1 PBJW (*r* = 0.19, *p* < 0.001), T1 GLS (*r* = 0.32, *p* < 0.001) and T2 altruism (*r* = 0.33, *p* < 0.001). T1 GBJW showed a significant positive correlation with T1 PBJW (*r* = 0.66, *p* < 0.001), T1 GLS (*r* = 0.37, *p* < 0.001) and T2 altruism (*r* = 0.41, *p* < 0.001). T1 PBJW was positively correlated with T1 GLS (*r* = 0.22, *p* < 0.001) and T2 altruism (*r* = 0.56, *p* < 0.001). T1 GLS was positively correlated with T2 altruism (*r* = 0.22, *p* < 0.001).

**Table 1:** Descriptive statistics and correlations between variables (*N* = 1764).

Variable	*M*	*SD*	1	2	3	4	5
1. T1 DSC	3.60	0.98	1				
2. T1 GBJW	4.53	1.09	0.05*	1			
3. T1 PBJW	4.50	1.05	0.19***	0.66***	1		
4. T1 GLS	4.51	1.13	0.32***	0.37***	0.22***	1	
5. T2 Altruism	3.70	0.86	0.33***	0.41***	0.56***	0.22***	1

*p < 0.05, ***p < 0.001. T1, measured at Time 1; T2, measured at Time 2; DSC, downward social comparison; GLS, general life satisfaction; PBJW, personal belief in a just world; GBJW, general belief in a just world.

#### 2.4.3 Mediation effect test

**Figure 2:**
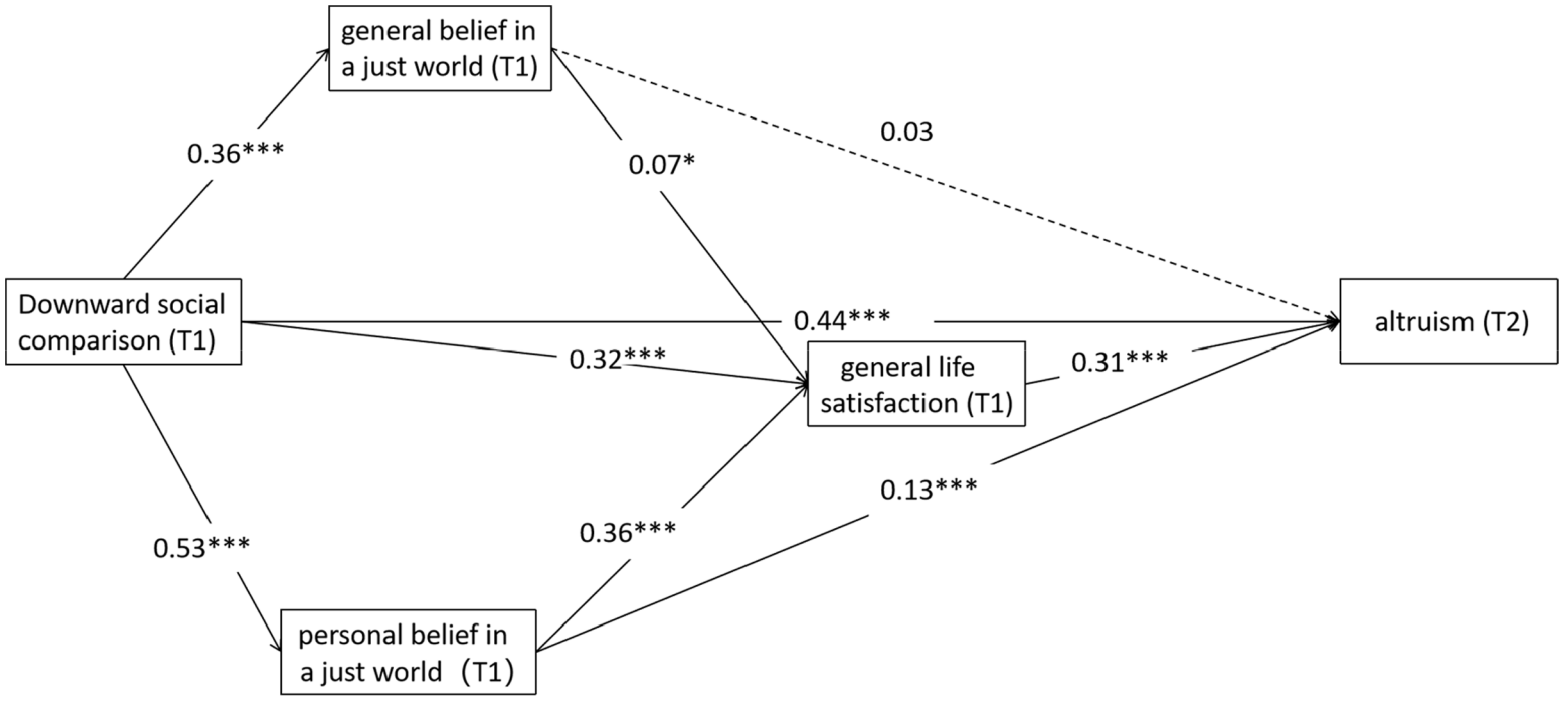
Multi-mediating model of DSC, GBJW/PBJW, GLS, and altruism. *p < 0.05, **p < 0.01, ***p < 0.001. T1, measured at Time 1; T2, measured at Time 2; DSC, downward social comparison; GLS, general life satisfaction; PBJW, personal belief in a just world; GBJW, general belief in a just world.

It can be seen from Table 1 that there is a significant pairwise positive correlation among all variables. Therefore, Mplus8.3 was used to further analyze the mediating effect of T1 GBJW, T1 PBJW, and T1 GLS based on controlling for demographic variables, and a structural equation model was constructed. The results indicate that the model has a good fit. CFI = 0.99, TLI = 0.98, *χ*^2^/df = 4.36, RMSEA = 0.04, SRMR = 0.02. The direct positive prediction of T2 altruism by T1 DSC was significant (*β* = 0.44, *p* < 0.001), so H1 was supported. T1 DSC positively predicted T1 GBJW (*β* = 0.36, *p* < 0.001) and T1 PBJW (*β* = 0.53, *p* < 0.001); T1 GBJW and T1 PBJW positively predicted T1 GLS (*β* = 0.07, *p* < 0.05; *β* = 0.36, *p* < 0.001); T1 GLS positively predicted T2 altruism (*β* = 0.31, *p* < 0.001), T1 PBJW positively predicted T2 altruism (*β* = 0.13, *p* < 0.001). No significant predictive effect of T1 GBJW on T2 altruism was shown (*β* = 0.03, *p* > 0.05) (see Figure 2).

The results of the analysis of mediating effects (see Table 2) show that T1 GBJW/T1 PBJW and T1 GLS play a multi-mediating role between T1 DSC and T2 altruism, with a mediating effect value of 0.24, accounting for 35.29% of the total effect of DSC on altruism (0.68). Specifically, the mediating effect consists of the indirect effects produced by four significant paths. Path 1: Indirect effects of T1 DSC → T1 GBJW → T1 GLS → T2 altruism (*β* = 0.01, *p* < 0.05); path 2: Indirect effects of T1 DSC → T1 PBJW → T2 altruism (*β* = 0.07, *p* < 0.001); path 3: Indirect effects of T1 DSC → T1 PBJW → T1 GLS → T2 altruism (*β* = 0.06, *p* < 0.001); path 4: Indirect effects of T1 DSC → T1 GLS → T2 altruism (*β* = 0.10, *p* < 0.001). The four indirect effects accounted for 1.47, 10.29, 8.82, and 14.71%, respectively, of the total effects.

**Table 2:** Results of the multi-mediating model.

Effect				95% conference interval	
	Intermediate path	Effect value	Boot standard error	Upper limit	Lower limit
Total effect	T1 DSC → T2 A	0.68***	0.02	0.73	0.64
Direct effect	T1 DSC → T2 A	0.44***	0.03	0.50	0.38
	T1 DSC → T1 GBJW → T1 GLS → T2A	0.01*	0.00	0.01	0.01
	T1 DSC → T1 PBJW → T2 A	0.07***	0.02	0.10	0.03
	T1 DSC → T1 PBJW → T1 GLS → T2A	0.06***	0.01	0.07	0.04
	T1 DSC → T1 GLS → T2A	0.10***	0.01	0.13	0.07

*p < 0.05, **p < 0.01, ***p < 0.001. T1, measured at Time 1; T2, measured at Time 2; DSC, downward social comparison; GLS, general life satisfaction; PBJW, personal belief in a just world; GBJW, general belief in a just world; A, altruism.

The data showed that T1 PBJW could independently and continuously mediate the influence of T1 DSC on T2 altruism in college students, together with satisfaction. Accordingly, H3 and H5 were supported. However, in this study, T1 GBJW did not significantly predict T2 altruism, so H2 was not supported. It was also found that PBJW played a stronger mediating role than PBJW, thus, H4 was not supported.

## 3 Discussion

This study explores the influence of DSC on altruism and the multi-mediating roles of PBJW, PBJW and GLS. The results of the correlation analysis showed that DSC was positively correlated with BJW, GLS, and altruism. These findings suggest that DSC is closely related to psychosomatic health and psychosocial adaptation (Helgeson and Taylor, 1993). The results of the mediating effect analysis further revealed that the mediating effect was produced through a direct path and four indirect paths: the independent mediating role of PBJW and GLS, multi-mediating of PBJW and GLS, GBJW and GLS. This study is consistent with previous research, suggesting that DSC not only directly affects individual altruism, but also indirectly affects altruism by mediating variables (Isen, 1970; Ding et al., 2018).

### 3.1 DSC and altruism

This study showed that DSC positively predicted college students’ altruism, which was consistent with H1. This suggests that college students who scored higher on the downward social comparison variable are more likely to engage in altruistic behavior. Warneken (2009) argued that social factors are important variables that influence altruistic behavior. As individuals grow older, they selectively choose to understand and experience altruistic behaviors based on their social cognition. This argument is supported by the results of this study, in which DSC was a significant positive predictor of altruism. This study is also consistent with existing research showing that DSC promotes individuals’ tendencies to be generous (Ye et al., 2021) and sympathetic toward those they are comparing themselves to, thereby exhibiting more apparent altruistic attitudes and behaviors (Batson, 1991). According to the empathy-altruism hypothesis, empathy has significant positive correlation with altruistic behavior, and the sympathy and compassion generated by empathy promote altruism (Baston, 2010). Based on the contrast-empathy model, we found that when compared to those who are less fortunate, people’s feelings of pity and empathy are awakened. However, other scholars believe that in the process of downward comparison, individuals, especially college students with strong self-awareness, tend to feel their worth and will enhance themselves by further empathizing with others or helping them, which will manifest as altruism in the outside world (Song et al., 2019).

### 3.2 The mediating effect of GBJW/PBJW

The mediating effect indicates that an independent variable on a dependent variable is transmitted through a third variable, which is called a mediator (VanderWeele and Vansteelandt, 2014). Our study found that BJW has a mediating effect between DSC and altruism. In other words, apart from direct effects, DSC also affects altruism through GBJW and PBJW (indirect effects). The social comparison theory states that people tend to compare themselves with others. This comparison impacts individual psychology and behavior, depending on the direction of the upward or downward comparison. Many studies believe that upward comparison has a negative impact on individuals (Wang et al., 2020) whereas downward comparison has a positive impact (Vrugt, 1994). This study confirms that this result also applies to the effects of social comparison on BJW. DSC can significantly and positively predict GBJW/PBJW and increases with an increase in DSC scores. By comparing downwards, individuals will gain a stronger sense of both GBJW and PBJW. This may explain why BJW is closely related to individuals’ social status. In DSC, individuals have a relative competitive advantage compared to individuals of a lower status or disadvantage, thus enhancing their BJW. In situations where individuals compare themselves to others with lower status or disadvantages, they may feel a sense of relative superiority and believe that they have earned their status through their efforts and deservingness, thus enhancing their BJW (Wang H. X. et al., 2021).

This study also found that PBJW could significantly and positively predict altruism, which indicates that college students with higher scores on PBJW are more likely to engage in altruistic behavior. BJW can enhance an individual’s sense of stability and control over the environment and promote the formation of altruism through the mediating role of gratitude and self-esteem (Jiang et al., 2017). In contrast to previous studies on collectivist cultures, in this study, PBJW showed a stronger mediating effect than GBJW. This may be related to the relationship between social comparison and altruism, which is the focus of this study. Bégue (2005) pointed out that PBJW plays a crucial role in helping individuals maintain their self-esteem when facing unfavorable social comparisons. Correia et al. (2016) showed that PBJW was significantly correlated with helping others, whereas GBJW was not. This may be due to the phenomenon of selfish bias in fair inference, referring to the tendency of individuals to perceive themselves as being more deserving of positive outcomes than others (Donat et al., 2018). This is possibly because the world itself is unjust, and there is a gap between the PBJW and GBJW. Some people are more likely to obtain justice than others (Thomas and Rodrigues, 2020). Therefore, we must acknowledge that when facing others less fortunate, people may not be motivated by their own desire to make the world fairer to everyone and bring happiness. However, their improved PBJW may encourage them to engage in altruistic behavior and demonstrate their superiority over those who are not as good as them.

### 3.3 The mediating effect of GLS

The results show that GLS has a significant mediating effect on DSC and altruism. This suggests that individuals who prefer to make DSCs are more likely to achieve higher general satisfaction, which can prompt them to engage in altruistic behavior. Downward comparison provides a low reference point for people to evaluate themselves, which helps them define their situation more positively, thereby improving their GLS (Buunk et al., 2001). Self-enhancement is one motive for DSC. In the process of downward comparison, individuals feel their value, their GLS improves, and they tend to further enhance themselves by empathizing with or helping others, which manifests as altruism in the outside world (Song et al., 2019). Therefore, when an altruistic situation exists, such as donating or volunteering, people can make downward comparisons. Moreover, when people seize this opportunity to compare their lives with assistance, they feel higher GLS, which motivates them to engage in altruistic behaviors (Huang, 2016).

### 3.4 The multi-mediating effect of BJW and GLS

Multi-mediating effect refers to multiple mediation with more than one intermediate mediator that has a series of indirect effects of dependent variables on independent variables (Robins and Greenland, 1992). Our results found that in the path of DSC affecting altruism, BJW and GLS showed a significant multi- mediating effect in the relationship between DSC and altruism; that is, DSC affected altruism by affecting BJW and GLS. DSC is more likely to maintain PBJW. It consists of the social comparison theory, in which unique patterns of cooperation among humans are based on the fairness of social comparison. People often prefer fairness and seek to reduce the unfairness between those who are superficially similar to them (Gheorghiu et al., 2021). Thus, individuals’ BJW affects their GLS (Tian, 2019) and behavioral patterns (Herrmann et al., 2019). When individuals feel that others are treated unfairly, BJW will make them think that they are favored by the world (Correia et al., 2016). When individuals obtain what they deserve, their GLS increases (Correia et al., 2009), and they are expected to maintain their inner BJW by helping others (Igou et al., 2021), showing their altruistic attitude and behavior (Duncanson, 2007; Gheorghiu et al., 2021). Based on the results of this study, during educational practice, intervention in social comparison among college students can enhance their perceived BJW, thereby improving their general life satisfaction and encouraging them to engage in more altruistic behaviors.

### 3.5 Limitations and future research

Though the current study has found some interesting things, it is still open for challenge or improvement because of some limitations. First, this study only considers the impact of downward comparison on altruism, while Li et al. (2021) suggested that downward comparison may not always bring positive effects, and the different mechanisms of downward comparison and downward identification should be considered. Similarly, the addition of upward comparisons, including upward contrast and upward identification, will enrich research results. Second, this study considered the causal relationship between downward comparison and altruism; however, the research subject was limited to college students. Future studies should increase the sample contrast between middle school and college students to reveal the dynamic relationship between downward comparisons and altruism more clearly. Finally, this study only discussed the influence of individual factors on altruism, which could increase the perspectives of society and family in the future, leading to a more comprehensive understanding of the development of altruism.

## 4 Conclusion

The Chinese proverb, “to fall short of the best but be better than the worst,” is often used to relieve the inner conflicts of individuals. Previous studies have also confirmed that DSC can actively promote individual mental health (Buunk and Dijkstra, 2017). This study explored the impact of a DSC perspective on individual altruism. We found that when college students were compared to individuals who performed worse than themselves, this actively promoted their altruism through the path of individual belief in a just world/general life satisfaction; the mediating effect of individual belief in a just world was stronger. These findings enrich the social comparison research and have significant implications for college students’ mental health and social development. They are also beneficial for educators in helping students learn to view and solve problems from multiple perspectives, thereby reducing developmental difficulties caused by thinking limitations. These findings enrich the social comparison research and have significant implications for college students’ mental health and social development. They are also beneficial for educators in helping students learn to view and solve problems from multiple perspectives, thereby reducing developmental difficulties caused by thinking limitations.

## Data availability statement

The raw data supporting the conclusions of this article will be made available by the authors, without undue reservation.

## Ethics statement

The studies involving humans were approved by The Institutional Review Board (or Ethics Committee) of Wenzhou University of Technology. The studies were conducted in accordance with the local legislation and institutional requirements. The participants provided their written informed consent to participate in this study.

## Author contributions

YH: Conceptualization, Data curation, Formal analysis, Funding acquisition, Investigation, Methodology, Project administration, Writing – original draft, Writing – review & editing. GC: Conceptualization, Data curation, Formal analysis, Investigation, Supervision, Writing – review & editing. LJ: Supervision, Writing – original draft. XL: Supervision, Writing – review & editing.
